# The association between self-rated health, number of family members, and cognitive function in community-dwelling older adults: Mediating role of depression

**DOI:** 10.1371/journal.pone.0306907

**Published:** 2024-07-09

**Authors:** Suyeong Bae, Yumi Ju, Sanghun Nam, Yeonju Jin, Sura Kang, Jeh-Kwang Ryu, Ickpyo Hong

**Affiliations:** 1 Department of Occupational Therapy, Graduate School, Yonsei University, Wonju-si, Gangwon-do, Republic of Korea; 2 Human Development and Rehabilitation, Graduate School of Education Service Science, Dongguk University, Seoul, Republic of Korea; 3 Convergence Research Center for Artificial Intelligence, Dongguk University, Seoul, Republic of Korea; 4 Department of Physical Education, College of Education, Dongguk University, Seoul, Republic of Korea; 5 Department of Occupational Therapy, College of Software and Digital Healthcare Convergence, Yonsei University, Wonju-si, Gangwon-do, Republic of Korea; St John’s University, UNITED STATES

## Abstract

With the increasing number of older adults, research on cognitive function has expanded. However, studies examining the mediating effect of depression on the association between complex factors and cognitive function in older adults are still insufficient. Additionally, there is a lack of studies that have investigated these relationships by integrating multiple factors related to the cognitive function of older adults. Therefore, our study investigated the association between the number of family members, self-rated health, depression, and cognitive function in community-dwelling older adults and highlighted the mediating role of depression in these relationships. We used data from 218 older adults aged over 65 collected in a previous study. The independent variables were the number of family members and self-rated health, and the dependent variable was cognitive function measured by the cognitive impairment screening test (CIST). The mediation variable was depression measured by the Patient Health Questionnaire-9 (PHQ-9). Structural equation modeling was used to examine the association between the independent, dependent, and mediation variable. The mean ages of the participants were 81.71 (standard deviation [SD] = 6.00) years, with 198 females (90.83%) and 20 males (9.17%). The structural equation model demonstrated a good model fit (chi-square value = 33.375; degrees of freedom = 24; *p*-value = 0.0964; RMSEA = 0.042; CFI = 0.970; TLI = 0.956; SRMR = 0.042). Self-rated health and the number of family members were not directly associated with cognitive function; however, depression had significant indirect effects (self-rated health to cognitive function: coefficient = −0.023, *p*-value = 0.017; number of family members and cognitive function: coefficient = 0.012, *p*-value = 0.030). Our findings indicated that depression plays a crucial mediating role between self-rated health, number of family members, and cognitive function. The results highlight the need for comprehensive strategies for mental health care to support cognitive health in older adults.

## Introduction

As the global population ages and the number and proportion of older adults continues to grow, cognitive functioning in aging adults has become a critical area of focus in many countries. Cognitive decline in older adults not only affects their quality of life but also has significant implications on their families and caregivers. Despite the growing attention to this set of issues, understanding specific factors that influence cognitive decline in this population remains limited. This gap in knowledge prevents the development of effective prevention and intervention strategies. For this reason, it is crucial to investigate the factors related to cognitive function to better address and prevent cognitive decline among older adults.

### Cognitive function and depression

Depression and cognition are associated with each other [1–4]. The relationship between depression and cognition in older adults is derived from older adults in many countries [1–3,5]. In Iran, older adults with severe depression symptoms had twice the risk for cognitive decline [3]. Furthermore, Kang et al. showed that depression is a risk factor that predicts the cognitive dysfunction in older Korean adults [5]. Beck’s cognitive model explains the interaction between depression and cognitive function, emphasizing that negative thinking patterns, biased attention, processing techniques, and memory induce and support depression, can lead to cognitive decline [6]. Disner studied the neurological mechanisms at play using Beck’s cognitive model, finding that depressive patients showed overactivity in the Default Mode Network and decreased activity in the Cognitive Control Network, related to cognitive decline [7].

### Cognitive function, self-rated health, and the number of families

The self-rated health is a factor that is correlated with cognitive decline. Previous studies have found that older adults having stable self-rated health have higher memory scores than those who do not have stable health. In addition, older adults with declining self-rated health tend to have greater memory loss than those with stable self-rated health [8]. Bond et al. (2006) found that older adults with worse self-rated health had a greater probability of cognitive impairment than those with higher self-rated health [9]. Thus, self-rated health is commonly indicated as a predictor of cognitive decline or the trajectories of cognitive function [8–10].

Another factor associated with cognitive function in older adults relates to the number of families. Previous researchers have reported the interaction between the number of family members and cognitive function in terms of social networks [9,11]. Fung et al. (2019) examined the association between social network size and cognitive function of older adults [11]. In that case, the size of the social network was defined as the a confiding network, family non-confiding network, and friends non-confiding network, based on the number of people in each group and whether one can tell their thoughts and feelings to them [11]. As a result, the interactions between the number of family members in the non-confiding network and loneliness were associated with cognitive function [11]. Moreover, older adults living alone showed more marked cognitive decline than older adults living with other families [12]. Another study suggested that the number of families is a social determinant of health, finding a positive association between the number of families and cognitive function in older adults [13].

### Depression, self-rated health, and number of families

Previous studies have reported that the self-rated health of older adults is associated with depression [9,14,15]. According to Ambresin et al. (2014), older adults who rated their health as bad had a higher risk ratio of major depression symptoms than those who evaluated that their health was good [15]. Additionally, self-rated health predicted major depression syndrome in adults and older adults for up to 5 years in a study [9].

The number of family members may be related to the living arrangements that can be linked with loneliness, depression, or social isolation, indicating the possibility of a relationship between the number of family members and depression. It has been reported that the number of family members is negatively associated with depression; a larger number of family members could lower the likelihood of developing depression and reduce the severity of depressive symptoms [16,17]. In Kim and Shin’s study (2020), a difference in depression was seen among older adults who received the services of long-term care insurance for the elderly about the number of family members, where the higher the number of family members, the lower the depression [18].

As mentioned above, previous studies showed possible association between self-rated health, depression, cognitive function and the number of family members. Despite these factors being significant for successful aging, there is unclear research about associations between these factors. In particular, self-rated health, depression, and cognitive function are associated with each other, although the relationships between these factors remains unclear [8,9,14,15]. Second, although the number of family members is related to cognitive function, the pathway to whether depression is related to cognitive function by being negatively related to the number of family members is yet unclear [1–4,9,11]. Therefore, our study aimed to investigate research hypotheses, including 1) self-rated health is associated with the cognitive function through depression, 2) the number of family members is associated with cognitive function through depression, and 3) the depression is associated with cognitive function.

## Materials and methods

### Study data and participants

Our study used the secondary data from previous research that was designed to develop a protocol for physical activity programs for the prevention of dementia (Previous study was approved by the institutional review board of Dongguk University (IRB No. DUIRB_202202–09). The data collection was conducted from July 29, 2021, to December 20, 2022. In the previous study, we obtained informed consent from the study participants. We accessed the data from June 1 to June 30, 2023, and the collected data included the demographics and clinical characteristics of 218 older adults aged over 65. Authors had no access to information that could identify individual participants during or after data collection.

### Study variables

#### Cognitive impairment screening test

We used the cognitive impairment screening test (CIST) to measure the cognitive function of older adults. It has been developed by the National Institute of Dementia of South Korea for application to the National Dementia Examination Project in South Korea [19]. The CIST comprised the following six subscales, including orientation, memory, attention, visuospatial, language, and execution functions with 13 items. The higher the score of the CIST means the better the cognitive function, and score has a range of 0 to 30 [20,21].

#### Self-rated health

We used a single question to assess self-rated health. The single question was “What do you think about your health”? It was measured by a 5-point Likert scale (1: very good, 2: good, 3: neutral, 4: bad, and 5: very bad). In the previous study, self-rated health was assessed by a single question, which is used by us [22–24].

#### Patient health Questionnaire-9

We used depression as a mediation variable. The symptoms of depression in the community-dwelling older adults were assessed by the Patient Health Questionnaire-9 (PHQ-9). The PHQ-9 was developed for the diagnosis of major depressive disorder based on the DSM-IV criteria. Each item was measured by a 4-point Likert scale (1: not at all, 2: several days, 3: more than half the days, and 4: nearly every day) according to the severity of depression symptoms [25]. We used the sum score of the PHQ-9, and the higher score means the severe depression symptom [25].

#### The number of family members

To use the number of family members as a variable, we used answer about the single question, “How many family members do the participants have in total”? The response was the number of family members.

### Statistical analysis

**Fig 1:**
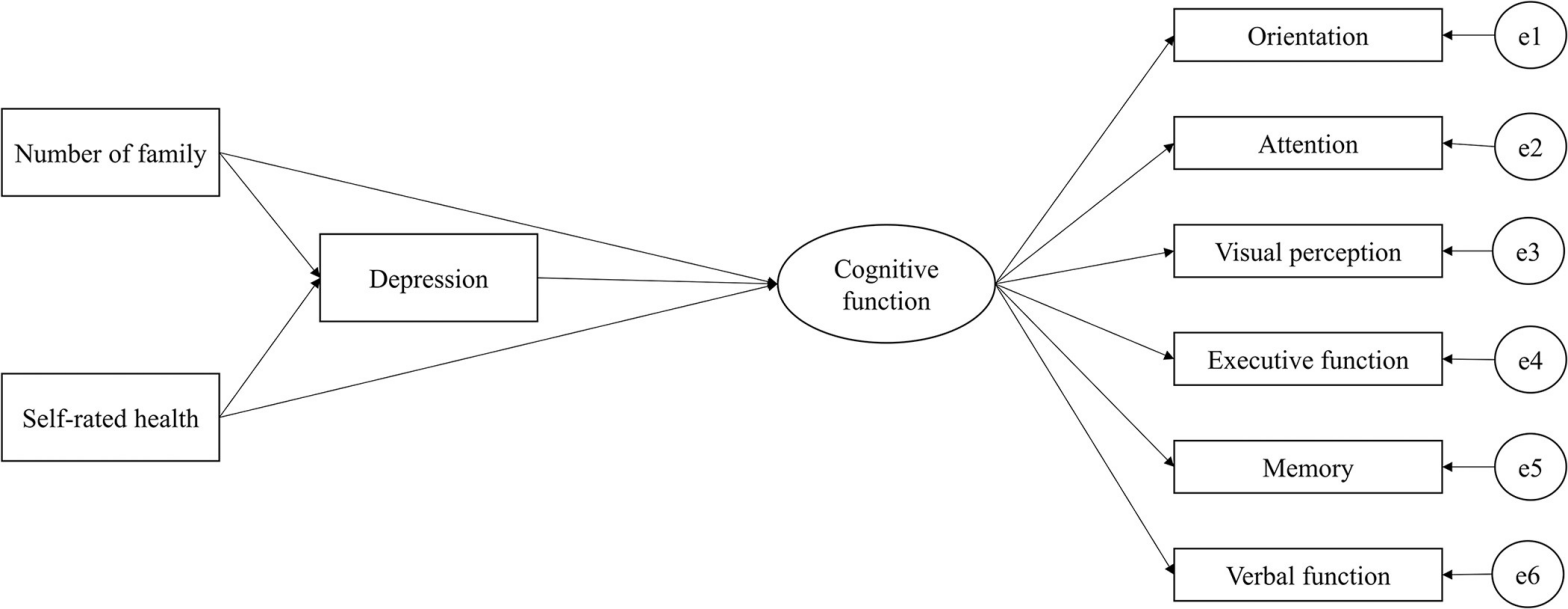
Conceptual model according to the research hypotheses.

We conducted structural equation modeling (SEM) to examine the association between self-rated health, depression, and cognitive function and the association between the number of family members, depression, and cognitive function. Initially, we analyzed the mean and standard deviation for continuous variables and the frequencies and percentile for categorical variables to investigate the demographic and clinical characteristics of the participants. Thereafter, we analyzed the uni-dimensionality of the CIST items by conducting the confirmatory factor analysis with one-factor model. The model fit criteria were applied as follows for deciding whether the model fitted: chi-square *p*-value > 0.05; root mean square error of approximation (RMSEA) < 0.05; confirmatory fit index (CFI) > 0.95; Tucker–Lewis Index (TLI) > 0.95; standardized root mean square residual (SRMR) < 0.1 [26,27]. Subsequently, we investigated the association between self-rated health, the number of family members, depression, and cognitive function which are represented in Table 1 by fitted those variables to the conceptual model (Fig 1). The SAS (version 9.4, SAS Institute, Cary, North Carolina, USA) was used for data management, and Mplus (version 8, Los Angeles, CA, USA) was used for conducting SEM.

**Table 1:** Demographic and clinical characteristics of the participants (N = 218).

Variables	*n* (%)
Age (mean ± SD^a^)	81.71 ± 6.00
Sex	
Female	198 (90.83)
Male	20 (9.17)
Marital status	
Have a partner	69 (31.65)
Not having a partner	149 (68.35)
Educational attainment	
Below elementary school	144 (66.06)
Middle school	39 (17.89)
High school	26 (11.93)
Above college	9 (4.13)
Chronic disease status (Yes)	
Myocardial infarction	26 (11.93)
Cerebrovascular disease	51 (23.39)
Number of family members (mean ± SD)	1.95 ± 1.21
PHQ-9^b^ (mean ± SD)	2.59 ± 3.20
Self-rated health (mean ± SD)	2.93 ± 1.26

^a^SD = standard deviation

^b^PHQ-9 = patient health questionnaire-9.

## Results

Table 1 presents the demographic and clinical characteristics of the participants. Their mean age was 81.71 (standard deviation [SD] = 6.00) years, and most of them were females (198, 90.83%). Among them, 69 (31.65%) had a partner, and 149 (68.35%) did not have a partner. The majority of the participants (144, 66.06%) had an academic level equivalent to or below elementary school. Table 2 shows the correlation matrix and mean value of the study variables.

**Table 2:** Correlation matrix of study variables.

	Orientation	Attention	Visual perception	Executive function	Memory	Verbal function	PHQ-9^a^	Number of family members	Self-rated health
Orientation	1.000								
Attention	0.346*	1							
Visual perception	0.300*	0.282*	1						
Executive function	0.412*	0.432*	0.436*	1					
Memory	0.458*	0.433*	0.285*	0.534*	1				
Verbal function	0.167*	0.271*	0.290*	0.335*	0.353*	1			
PHQ-9	−0.134*	−0.083	−0.0254*	−0.140*	−0.041	−0.019	1		
Number of family members	0.043	0.035	−0.029	0.002	−0.005	0.076	−0.148*	1	
Self-rated health	−0.028	0.022	−0.095	0.006	0.030	−0.068	0.273*	−0.054	1
Mean	4.317	2.179	1.413	3.523	8.078	3.216	2.592	1.950	2.927
SD^b^	1.028	0.644	0.861	1.434	2.347	0.675	3.203	1.208	1.257

^a^ PHQ-9 = patient health questionnaire-9

^b^SD = standard deviation

*p < 0.05.

**Fig 2:**
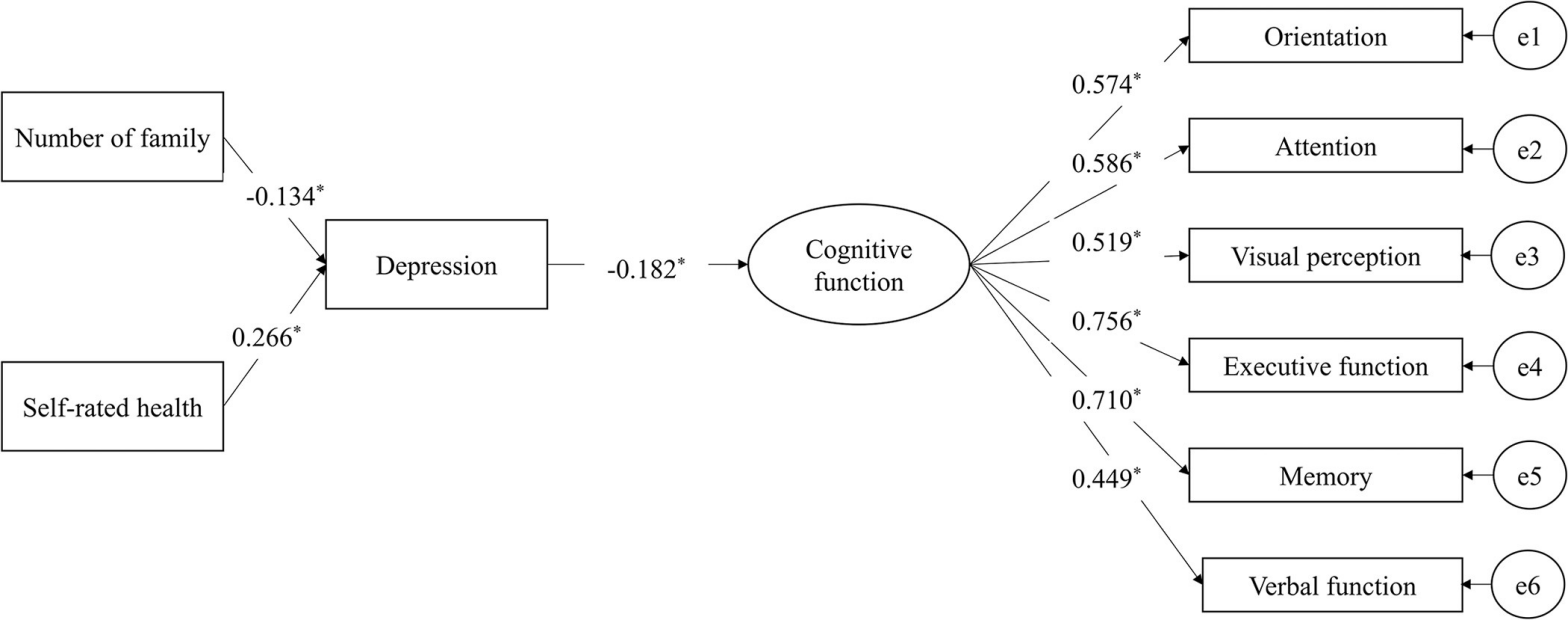
Path coefficients and standardized factor loadings in the CIST measurement model. *p < 0.05.

Fig 2 shows the results of the confirmatory factor analysis of the CIST, and the path coefficients of the study variables. The model fit result yielded that the measurement model of the CIST had a good fit to the data, with all factor loadings being above 0.4 (lambda range: 0.453–0.753, all *p* < 0.05; chi-square value = 14.065, degrees of freedom [df] = 9, *p*-value = 0.12; RMSEA = 0.051, CFI = 0.982, TLI = 0.970, SRMR = 0.031).

Table 3 displays the unstandardized and standardized coefficients of each path. The model fit value indicated that the model fits the data (chi-square value = 33.375, df = 24, *p*-value = 0.0964; RMSEA = 0.042; CFI = 0.970; TLI = 0.956; SRMR = 0.042). The number of family members was associated with depression (standardized coefficient = −0.134, *p*-value = 0.037) but not cognitive function (standardized coefficient = −0.003, *p*-value = 0.964). Similarly, there was an association between self-rated health and depression (standardized coefficient = 0.266, *p*-value < 0.000) but not cognitive function (standardized coefficient = 0.035, *p*-value = 0.651). Depression was associated with cognitive function (standardized coefficient = −0.182, *p*-value = 0.019). There is an indirect effect of depression between self-rated health and cognitive function (standardized coefficient = −0.048, *p*-value = 0.017), as well as between the number of family members and cognitive function (standardized coefficient = 0.024, *p*-value = 0.030).

**Table 3:** Path coefficients in the conceptual model.

Path	Unstandardized coefficient (95% CI)	Standardized coefficient (95% CI)	*p*-value
Number of family members → Depression	−0.355 (−0.625, −0.106)	−0.134 (−0.216, −0.041)	0.037*
Number of family members → Cognitive function	−0.002 (−0.076, 0.075)	−0.003 (−0.140, 0.145)	0.964
Self-rated health → Depression	0.677 (0.397, 0.937)	0.266 (0.161, 0.380)	0.000**
Self-rated health → Cognitive function	0.017 (−0.064, 0.084)	0.035 (−0.144, 0.179)	0.651
Depression → Cognitive function	−0.034 (−0.066, −0.010)	−0.182 (−0.300, −0.061)	0.019*
Self-rated health → Depression → Cognitive function	−0.023 (−0.045, −0.006)	−0.048 (−0.092, −0.015)	0.017*
Number of family members → Depression → Cognitive function	0.012 (0.002, 0.023)	0.024 (0.005, 0.048)	0.030*

*p < 0.05

**p < 0.01.

## Discussion

The present study investigated 1) the association between self-rated health and cognitive function through the mediation effect of depression, 2) the association between the number of family members and cognitive function through the mediation effect of depression, 3) the association between depression and cognitive function. Our findings indicate that depression has a mediation effect between self-rated health and cognitive function and between family members and cognitive function. It represents that depression is an important part of older adults for cognitive function from self-rated health and number of family members.

Depression plays a significant role in the relationship between self-rated health and cognitive function. Self-rated health is subjective and perceived health status of the individual, and it is strongly related to one’s objective health condition and mortality [28]. It has been reported that self-rated health is highly related to psychosocial factors such as social isolation, negative life events, depression, and stress [29]. Our study provided further evidence that depression has a mediating effect between self-rated health and cognitive function, meaning that depression is a within-person factor that can modulate cognitive health [29,30]. The number of older adults experiencing depressive symptoms is increasing worldwide [31]. This rising trend, combined with our results, indicates that older adults with depressive symptoms face more than emotional difficulties, including cognitive decline, which may be influenced by depressive symptoms. Depression may be a common occurrence in aging process, but it should be monitored and intervened by health professionals at an early stage for their long-term well-being [32,33]. Additionally, it is necessary to manage older adults’ depression for their health from a national system-level perspective either [34–37].

The mechanism of association between self-rated health and cognitive decline is unclear; however, our results are evidence that can help interpret this mechanism. Previous studies have reported that self-rated health can predict cognitive decline or dementia in older adults [9,38]. However, these causal or associative mechanisms have not been explained. Our results can identify these association mechanisms. Pessimistic and negative perceptions of health can distort the perception of one’s own health and increase vulnerability to future diseases [29]. In addition, self-rated health also reflects health beliefs and behaviors; thus, negative beliefs and behaviors may be associated with cognitive decline [38]. Several studies have suggested that depression can be included in this process, especially since depression is a contributing factor in increasing the risk or incidence of Alzheimer’s disease, so it is important to identify its role in this process [4,29,39]. Although previous studies have suggested a possible relationship between self-assessed health, depression, and cognitive function, this relationship has not been comprehensively investigated [8,15,40]. The model presented in our study was designed to compensate for this gap in previous studies, and our results indicate the mediation effect of depression between self-rated health and cognitive function. These results can be extended to create several models explaining between self-rated health and cognition.

Our study found a mediating effect of depression between the number of family members and cognitive function; however, no direct association appeared between the number of family members and cognitive function, indicating that that depression may have mediated the relationship between the number of family members and cognitive function reported by previous studies. In other words, the higher the number of family members, the lower the level of depression in older adults, leading to an extended result of prevention of cognitive decline. Our findings can be explained by the loneliness of older adults. The depression of older adults is becoming a social issue in modern society, where family forms, such as the loss of a spouse and not living with children, are increasingly becoming a single-person household, and communication between families is decreasing [41]. Holvast et al. (2015) showed that older adults with more severe loneliness were associated with poor prognosis of depression 2 years later [42]. Moreover, Chao (2011) reported similar results, assessing the proximity of support of older adults according to whether they lived with a married son or not; that study found that the proximity of support was associated with depression [43]. Accordingly, our results, combined with earlier findings, indicate that the lower number of family members living with older adults is associated with cognitive decline as it causes loneliness and increases depressed emotions. Thus, creating social network around older adults’ individual could prevent some cognitive decline and enable aging in place for older adults.

It is necessary to consider the ways to alleviate the depression of older adults live alone or with small family members in the community or have bad self-rated health. Many studies report represent that physical activity can decrease probability of depression of older adults who live alone. Lee et al. (2022) reported that depressive symptoms decreased as a result of exercise intervention in older adults living alone [44]. Similarly, another study showed that regular walking activity was associated with decreasing depression symptoms in older adults living alone [45]. These effects can be explained by the mechanism that physical activity can activate neuroplasticity that is related to depression [46]. In addition, the effects of physical activity on the depression of older adults who have poor self-rated health are unclear; however, physical activity can affect depression in older adults who have poor self-rated health as shown in reports of a positive association between physical activity and self-rated health. Generally, the aim of providing exercise for the older adults is usually to increase overall health or physical function, but our results also show that the effect of exercise on the emotional and cognitive health of older adults is also important.

Another way to prevent depression is to have a good neighborhood environment. The neighborhood environment can be made up of physical factors like park accessibility and social factors like close neighbors [47]. These are associated with decreasing the depression of older adults who live with family or alone [47,48]. Zhang et al. (2019) showed that facilities for pedestrians can cause increased physical activity in older adults, and it is associated with decreasing depression [48]. Further, the neighborhood social environment was associated with depression in older adults living alone [47]. The physical and social environments of the neighborhood may be related to the reduction of depression in older adults, but the specific impact of these on other health factors by reducing depression is not yet clear. Therefore, in future studies, it is necessary to investigate the clear relationship between the physical and social environment creation of neighbors with the health factors of older adults, focusing on the mediating effect of depression.

Since 2008, the Korean government has implemented the National Dementia Plan, including a prevention program for healthy older adults and cognitive activity program for those with mild cognitive impairment [49]. Additionally, a management program called the Specialized Service in the Individualized Support Service is being provided for depressed older adults living in the community [50]. However, these programs and policies are currently being implemented separately. Our results suggest that a closer connection between policy practitioners and program practitioners is necessary for these programs. Our findings reveal a negative association between depression and cognitive function. In particular, depression mediates the relationship between self-reported health, the number of family members, and cognitive function. Therefore, it is essential to continuously monitor factors negatively affecting cognitive function in older adults, including, particularly, those reporting poor health, those living alone with limited family support, and those suffering from depression.

Our study has some limitations. First, we used self-rated health using a single question. A single question has the advantage of the rapidity of the survey, but it may not be enough to represent one factor. This limitation is significant, as the reliability and validity of single-item measures can undermine the rationality of the mediation model. Furthermore, the number of family members was also used as a single item, which may not capture the quality or nature of family relationships that impact cognitive function and depression. Therefore, future studies should include comprehensive measures that can more accurately represent self-rated health and family dynamics. By incorporating multi-item scales and ensuring their validity, future research should provide a more robust analysis of the mediating effect of depression on cognitive functions. Second, our study participants lived only in metropolitan cities. Our results would not be generalized to other populations because older adults in metropolitan cities and older adults in rural areas have different characteristics. Future research should collect information on older adults in rural areas, and analyze the differences between metropolitan cities and rural areas in a multi-level analysis. Third, we could not consider possible confounding variables, such as physical health, socioeconomic status, or the quality of social support networks beyond the family structure, that could have influenced the results due to our reliance upon secondary data. Thus, future studies must sharpen the expanded mediation role of depression while controlling confounding variables.

## Conclusions

Our study found that depression mediates the association between self-rated health, the number of family members, and cognitive function in community-dwelling older adults. Specifically, there were indirect effects of depression between self-rated health and cognitive function, as well as between the number of family members and cognitive function. These findings highlight the connecting role of depression in association with these variables. Therefore, health professionals need to understand and address depressive symptoms to help prevent cognitive decline in community-dwelling older adults. In addition, our results could be evidence of the importance of depression in preventing cognitive decline in community-dwelling older adults.

## Supporting information

S1 Table Correlation matrix, mean, and standard deviation of study variables. (DOCX)
