# Supplementary material for: The association between self-rated health, number of family members, and cognitive function in community-dwelling older adults: Mediating role of depression
Source: PLoS One. 2024 Jul 9;19(7):e0306907. doi: 10.1371/journal.pone.0306907 (PMC11232972; doi:10.1371/journal.pone.0306907)
Supplement: S1 Table — (DOCX) [file pone.0306907.s001.docx]

**S1 Table. Correlation matrix, mean, and standard deviation of study variables**

| Variables | Total score of CIST^a^ | PHQ-9^b^ | Number of family members | Self-rated health |
| --- | --- | --- | --- | --- |
| Total score of CIST^a^ | 1.0000 |  |  |  |
| PHQ-9^b^ | -0.1465^*^ | 1.0000 |  |  |
| Number of family members | 0.0089 | -0.1483^*^ | 1.0000 |  |
| Self-rated health | -0.0141 | 0.2729^*^ | -0.0540 | 1.0000 |
| Mean | 22.716 | 2.592 | 1.950 | 2.927 |
| SD^c^ | 5.069 | 3.203 | 1.208 | 1.257 |

^a^CIST = cognitive impairment screening test; ^b^PHQ-9 = patient health questionnaire-9; ^c^SD = standard deviation; ^*^*p*<0.05
